# Partial deligandation activated ZIF-67 for efficient electrocatalytic oxygen reduction reaction

**DOI:** 10.3389/fchem.2022.983549

**Published:** 2022-10-06

**Authors:** Xiaoli Yang, Jiali Gu, Chenhong Liu, Zhengyu Bai, Lin Yang

**Affiliations:** Collaborative Innovation Center of Henan Province for Green Manufacturing of Fine Chemicals, Key Laboratory of Green Chemical Media and Reactions, Ministry of Education, School of Chemistry and Chemical Engineering, Henan Normal University, Xinxiang, China

**Keywords:** ZIF-67, activation, deligandation, low temperature, oxygen reduction reaction (ORR)

## Abstract

Removing the blocked molecular groups and fully exposing the intrinsic active sites of metal-organic frameworks (MOFs) could give full play to their advantages of multi-active sites and multi-channel mass transfer, which will benefit the electrocatalytic oxygen reduction reaction (ORR) in fuel cells. Here, the partial diligandation-activated ZIF-67 (named as ZIF-67–400) with excellent ORR performance was obtained by simple low-temperature pyrolysis. The ORR electrocatalytic activity exhibits a half-wave potential of 0.82 V and the stability of maintaining 96% activity after 10 h of operation, which is comparable to commercial Pt/C. Further research studies reveal that the morphology, special dodecahedron configuration, and crystal structure of ZIF-67-400 are maintained well during the pyrolysis, but some hydrocarbon groups in the ligands are eliminated, resulting in the active sites being exposed and coordinated with the intrinsic porosity, improving the catalytic performance. This work may provide an alternative path for activating the electrocatalytic performance of metal-organic frameworks by low-temperature annealing.

## 1 Introduction

During the exploration of new types of power equipment, fuel cells and metal air cells have attracted much attention due to their sustainability, environmental friendliness, and high capacity (Dunn et al., 2011; Cheng and Chen, 2012; Chu and Majumdar, 2012; Wang et al., 2021a). The oxygen reduction reaction (ORR) is an important process for energy conversion and storage in fuel cells and metal air cells. Unfortunately, the ORR is affected by the inherent slow kinetics due to its complex multi-electron reaction pathway (Katsounaros et al., 2014). Although platinum-based materials are considered to be the most effective catalysts for the ORR to reduce overpotential (Jiao et al., 2015; She et al., 2017; Sun et al., 2021), the high price and poor stability of platinum-based catalysts severely limit their large-scale application. Therefore, the focus of fuel cells and metal air cells is to develop non-precious metal catalytic materials with excellent ORR activities and long-term stability, which is conducive to meeting the needs of new energy sources (Xia et al., 2016; Liu et al., 2017; Najam et al., 2018; Najam et al., 2020).

Among the non-precious, metal-based electrocatalysts for the ORR, metal-organic frameworks (MOFs) and their derivatives have attracted tremendous attention because of their high surface areas, tunable pore structure, and intrinsic metal sites (Zhang et al., 2017a; Jiao et al., 2017; Lu et al., 2021). Most research show that the superior catalytic performance of MOF derivatives is related to their metal–nitrogen–carbon (M-N-C) structures formed during the calcination of MOFs (Chung et al., 2017; Ding et al., 2019; Shah et al., 2020; Zhou et al., 2021; Wan et al., 2022); especially for the single-atom catalyst with the best performance at present within non-precious metal catalysts, its effective active site has been proven to be M-N_4_ structure (Shah et al., 2018a; Shah et al., 2021a; Wang et al., 2021b; Shah et al., 2022a). It is shown that the formation of these derivatives often needs to be calcined at a higher temperature, which will lead to greater energy consumption and environmental pollution and the collapse of the parent MOF skeleton (Shah et al., 2018b; Hu et al., 2021; Shah et al., 2022b). Considering the abundant active sites and variable pore structure in MOF skeletons, how to expose the active sites while maintaining their porosity without high temperature calcination is worth exploring. In this work, a new strategy of exploring the intrinsic M-N_x_ coordination active sites in MOFs with their abundant porosities for the electrocatalytic ORR without high energy consumption is proposed. ZIFs (zeolitic imidazolate frameworks) are a series of MOFs with a zeolite-like framework and intrinsic M-N_4_ active sites (Phan et al., 2010; Zhang et al., 2017b; He et al., 2019), which is beneficial to the ORR process and is suitable to be used as a model to explore the exposure of the intrinsic active sites. The popular Co-based ZIF (ZIF-67) has been studied extensively in the ORR, OER (oxygen evolution reaction), and other electrocatalytic processes by high-temperature pyrolysis to its derivatives and exhibited efficient catalytic performance (Ma et al., 2011; Zhao et al., 2014; Shah et al., 2017; Najam et al., 2019; Shah et al., 2021b; Imran et al., 2022), but the catalytic activity of the parent ZIF-67 is poor. In other words, the intrinsic active sites in ZIF-67 are unable to be exposed to promote the catalytic activity under such conditions and need to be activated. One possible reason for this low exposure of metal sites is the blocking ligands around the metal ions. So, removing the ligands to some extent without changing the MOF’s skeleton is a probable method, such as plasma etching (Tao et al., 2017), post-synthesis thermal treating (Gadipelli et al., 2014; Gadipelli and Guo, 2014; Chen et al., 2022), and low-temperature etching (Guo et al., 2019a; Zhu et al., 2020).

In this study, we show a simple and general method to increase ORR active sites and enhance their catalytic activity by using low temperature and creating surface defect structures. The results exhibited that the activated ZIF-67 showed comparable activities with other M-N-C materials without high-temperature pyrolysis and MOF disruption. The key to this annealing process is to control the heat treatment temperature close to the skeleton decomposition temperature for local modification to create the surface defect structure so as to increase the active area and active sites of the catalyst. This work may offer some new thinking for modifying MOFs as activated ORR electrocatalysts at low temperature.

## 2 Experimental section

### 2.1 Synthesis of the sample

Materials: All chemical reagents were of at least analytical grade or higher and were used as purchased without further purification. 2-Methylimidazole was purchased from American Aladdin company, Co(NO_3_)_2_·6H_2_O was obtained from Beijing Chemical and Engineering Company, methanol and ethanol were purchased from Tianjin Deen Chemical Reagent Co., Ltd., KOH was acquired from Tianjin Tianli Chemical Reagent Co., Ltd., Nafion was purchased from Shanghai Hesen Electric Co., Ltd., and N_2_, O_2_, and NH_3_/Ar were purchased from Beijing Praxair Utility Gas Co., Ltd.

The synthesis of ZIF-67 was slightly modified by the previously reported method as follows (Tao et al., 2017): 0.985 g of 2-methylimidazole was dissolved in 30 ml of methanol and 0.291 g of Co (NO_3_)_2_·6H_2_O was dissolved in 10 ml of methanol, and then the two solutions were mixed and stirred for 24 h at room temperature. The product was obtained by centrifugation, washed with methanol, and dried in vacuum. The prepared ZIF-67 was put into a tubular furnace and heated at different temperatures (200, 300, 380, 400, and 420°C) in a nitrogen atmosphere for 2 h, cooled to room temperature, and the products of ZIF-67-200, ZIF-67-300, ZIF-67-380, ZIF-67-400, and ZIF-67-420 were obtained.

### 2.2 Physical characterization

X-ray diffraction (XRD) results were analyzed on a PANalytical X’Pert Pro Diffractometer assembled with Cu–Ka radiation (λ ¼ 1.5418 A) from 10° to 80° (2θ). Field emission scanning electron microscopy (FESEM) observations were conducted on a JEOL JSM-6701F at an accelerating voltage of 15.0 kV. Transmission electron microscopy (TEM) was operated using a JEOL-100CX at an operating voltage of 100 kV. High-angle annular dark-field scanning TEM (HAADF-STEM) images were performed using a Hitachi aberration-cORRected STEM HD-2700C. X-ray photoelectron spectroscopy (XPS) spectrums were performed on a Thermo Scientific Escalab 220i-XL. The specific surface areas were evaluated by Micromeritics ASAP 2020. Raman spectrums were obtained on a HORIBA FRANCE SAS (LabRAM HR Evolution) with a 532-nm excitation length. Thermogravimetric (TG) analysis was carried out on a thermal analysis instrument (STA449C) in an air flow within a temperature range of 25–1,000°C at a heating rate of 10°C min^−1^.

### 2.3 Electrochemical measurements

All electrochemical measurements were performed at the Shanghai Chenhua Electrochemical Workstation CHI 760e, using a saturated calomel electrode (SCE) as a reference electrode, a carbon rod as a counter electrode, and a glassy carbon rotating disk electrode as a working electrode.

The working electrode was prepared by the following steps: 2 mg of the catalyst was ultrasonically dispersed in the mixture solution of 485 μl ethanol and 15 μl Nafion solution to obtain the catalyst slurry solution. The catalyst slurry was transferred to the GC electrode and dried at room temperature. Linear sweep voltammetry (LSV) and cyclic voltammetry (CV) were performed at 5 mV s^−1^ scan rate in a 0.1-M KOH electrolyte saturated with O_2_ or N_2_.

For the ORR at an RRDE, the electron transfer number (n) and the hydrogen peroxide yield (H_2_O_2_%) were obtained by equations:

$$n=4\times \frac{I_{d}}{I_{d}+\frac{Ir}{N}},\;H_{2}O_{2}\left(\%\right)=200\times \frac{\frac{Ir}{N}}{Id+\frac{Ir}{N}},$$

where I_d_ and I_r_ are the disk current and the ring current, respectively, and the current collection efficiency of the Pt ring is 0.37.

## 3 Results and discussion

**FIGURE 1:**
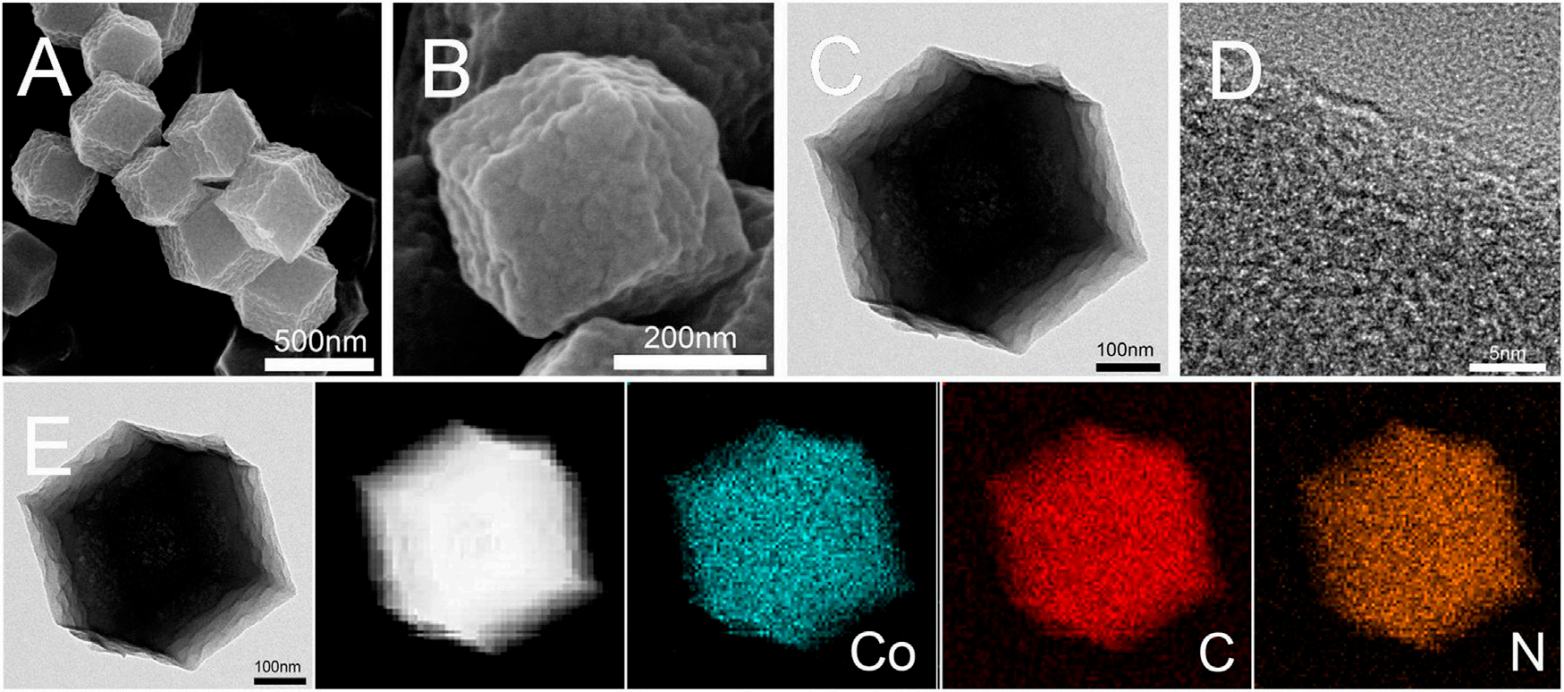
FESEM and TEM images of the ZIF-67-400 catalyst: **(A,B)** FESEM images with different magnification, **(C,D)** TEM images and **(E)** mapping images.

ZIF-67-400 nanocrystals were prepared by stirring the precipitate in methanol solvent at room temperature and heating treatment at 400°C. The morphology and composition of the catalyst were studied by FESEM and TEM, respectively (Figure 1). ZIF-67-400 retained the morphology of the ZIF-67 precursor (Figures 1A–C), showing a polyhedral structure, but the surface of the catalyst was wrinkled after local pyrolysis. Moreover, the particle size of the catalyst is slightly smaller than that of the precursor because the organic carbon skeleton shrinks during high-temperature treatment. The element diagram in Figure 1E shows the uniform distribution of Co, C, and N in the polyhedral carbon skeleton, which is consistent with the precursor ZIF-67 without local pyrolysis.

**FIGURE 2:**
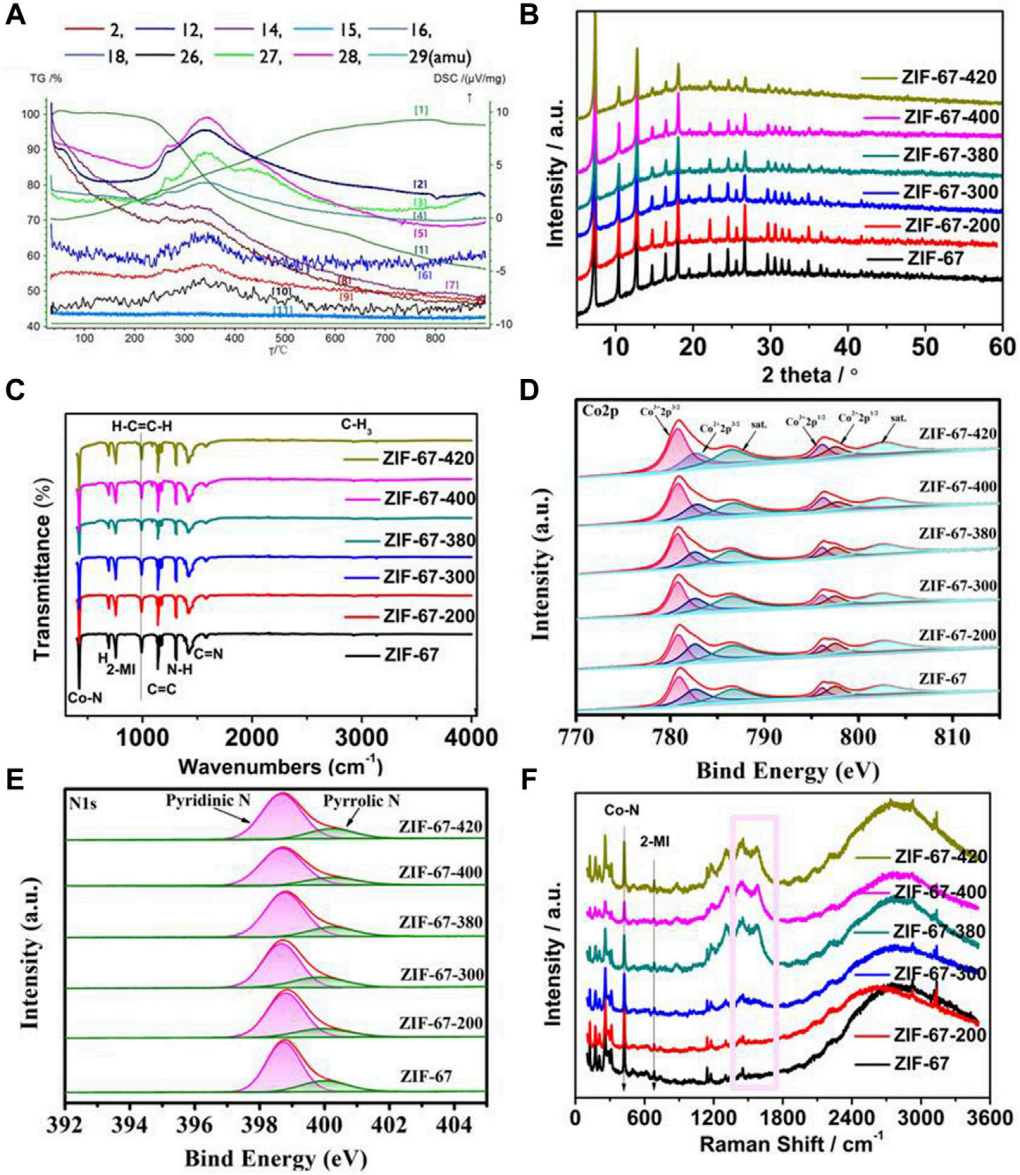
**(A)** TG-MS of the ZIF-67-400 catalyst (The curves labeled [1-11] refer to the overall Thermogravimetric curves and TG-MS curves attributed to amu 18-H2O, amu27-HCN, amu29-H2CNH, amu28-N2/H2CN, amu12-C, amu14-CH2, amu16-CH4, amu2-H2, amu26 C2H2, and amu15-CH3, MS signals in the atomicmass unit (amu) represent the evolution of decomposed gaseous species during TG analysis.), **(B–F)** are the XRD, FT-IR, Co 2p, N 1s, and Raman spectra at different heating temperatures (the pink block diagram in F represents the D-band and G-band of the carbon material).

As shown in Figure 2A, under the inert atmosphere with a continuous heating rate of 2°C per minute, the thermogravimetric analysis and mass spectrometry (TG-MS) of ZIF-67 showed that the skeleton began to decompose or carbonize at about 300°C, which was consistent with the earlier report. With the increase of temperature above 300°C, ZIF-67 decomposes locally and shows decomposition and mass loss due to volatilization of H_2_ and the release of hydrocarbons and C-N-H species (Gadipelli and Guo, 2014). At 400°C, the loss reaches its maximum. It exhibited that by controlling the calcination temperature near but below the complete-carbonization temperature, the local-dissociation in the skeleton will occur, which provides ZIF-67 with interesting properties. The carefully controlled heating process after synthesis will gradually change the bright purple ZIF-67 into a dark purple material.

As shown in Figure 2B, the X-ray diffraction (XRD) patterns of the samples after heat treatment support the TG-MS results. Most of the rhomboidal dodecahedral structures of ZIF-67 are retained at 400°C. The sharp XRD peaks at low angles imply the long-range ordered structure to a large extent, while the decrease of XRD peak intensity at high angles indicates the existence of local short-range disorder. Meanwhile, the infrared spectrum in Figure 2C shows that the thermal-treated sample still retains the framework structure of ZIF-67, and the main structural units of ZIF-67 remain unchanged with the removal of some groups from the ligand.

X-ray photoelectron spectroscopy (XPS) was used to further investigate the local structural bonding environment in the heated ZIF. The XPS spectra of Co 2p in Figure 2D and N 1s in Figure 2E exhibited that the calcinated ZIF-67 at different temperatures has a similar energy band compared to the parent ZIF-67, which means that the element valence and chemical bond are unchanged. The deconvoluted Co 2p spectra of ZIF-67 and the calcinated ZIF-67 in different temperatures in Figure 2D were split into Co^3+^ 2p_3/2_ (780.7 eV) and Co^2+^ 2p_3/2_ (782.6 eV) and Co^3+^ 2p_1/2_ (796.1 eV) and Co^2+^ 2p_1/2_ (797.4 eV) accompanied by satellite peaks at 786.4 and 802.6 eV, respectively. The high-resolution N 1s spectra of samples (Figure 2E) display two main peaks for pyridinic-N (398.7 eV) and pyrrolic-N (400.2 eV) (Gadipelli et al., 2014; Liu et al., 2021; Meng et al., 2022; Qi et al., 2022; Zhang et al., 2022). It can be seen from Supplementary Figure S1A that the content percentage of pyridinic N and pyrrolic N changes from ZIF-67 to the calcinated ZIF-67 in different temperatures. From ZIF-67 to ZIF-67-400, the content of pyridinic N increases and the content of pyrrolic N decreases, which implies the pyridinic N is attributed to enhancing the electrocatalytic activities of ZIF-67-400. As shown in Supplementary Figure S1B, the ratio of C:N:Co in ZIF-67-400 is lower than that in ZIF-67, which is consistent with the partial ligand loss during the calcination and is in line with the best catalytic activity of ZIF-67-400, superior to other samples in other temperatures and the pristine ZIF-67. The decrease of C:N:Co suggests that the partial ligand loss occurred in gentle calcination generates exposed coordinatively Co-N_x_ sites, which is responsible for ORR activity.

Figure 2F is the Raman spectrum of the sample. The peak in the wavelength within 1,000 cm^−2^ is due to the metal-bond and some functional groups, such as Co-N and methylimidazole. The peaks located within 1,100 cm^−2^ to 1,600 cm^−2^ indicate the degree of graphitization and defects in the structure (the pink block diagram in Figure 2F) (Zhu et al., 2020; Wang et al., 2022). From the parent ZIF-67 to the calcinated ZIF-67, it can be observed that the intensity and ratio of these peaks increase in turn, indicating that the samples gradually show graphitization and defects, which corresponds to the aforementioned thermogravimetric analysis and XPS data.

All the aforementioned experimental data show that some groups of hydrocarbons and C-N-H species dissociation occur in the heated ZIF, while the whole framework remains intact. We notice that the relatively weak chemical bonds in the ZIF-67 crystal are the Co–N coordination bond and the C–C bridge bond between the methylimidazole ligand and the methyl group, while the other bonds in the imidazole ligand have strong bond energy and are not easy to break. In the process of increasing temperature, the skeleton began to fracture naturally at the junction of Co–N and C–C. It is worth noting that each Co is coordinated with 4 N atoms and located in the limited space, while the terminal-CH_3_ group is free, so it is easier to fall off, leading to the formation of defects.

**FIGURE 3:**
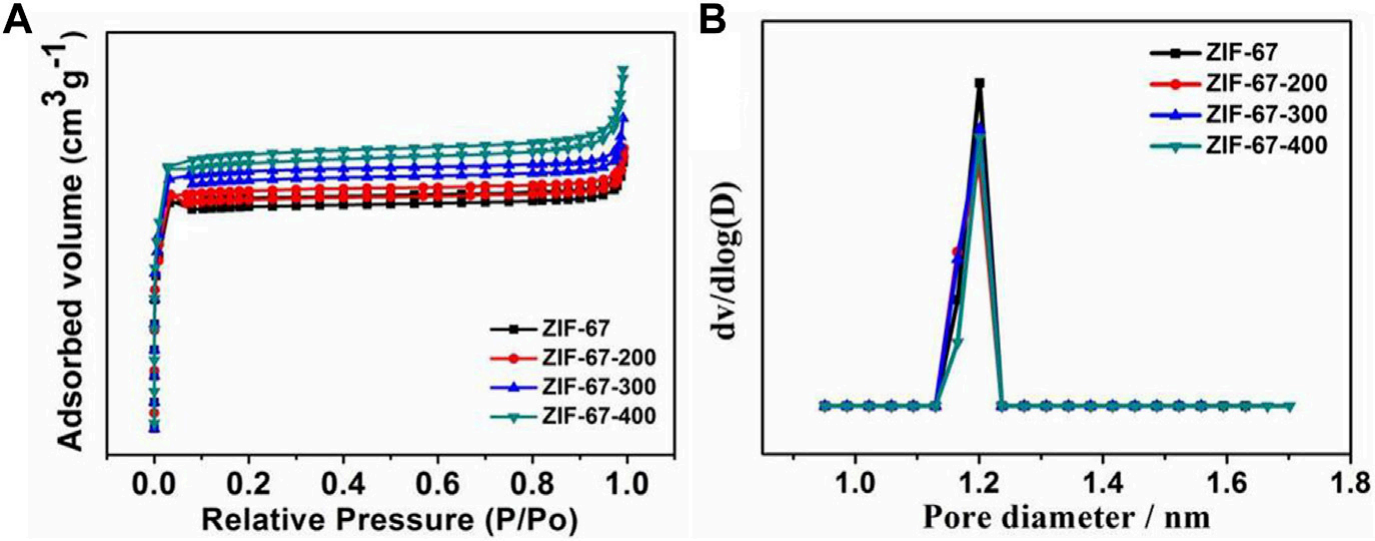
**(A,B)** N_2_ isothermal adsorption–desorption curve and pore size distribution of ZIF-67 at different pyrolysis temperatures.

In order to further understand the structure of ZIF-67 after heating, the porosity of samples was measured by the N_2_ isothermal adsorption–desorption curve. As shown in Figure 3, the N_2_ adsorption–desorption isotherm and the corresponding pore size distribution results show that from the pristine ZIF-67 to the calcinated samples, the skeleton structure has almost no change with the almost same BET isothermal curve and pore size distribution, which is very consistent with the FT-IR and XPS data.

**FIGURE 4:**
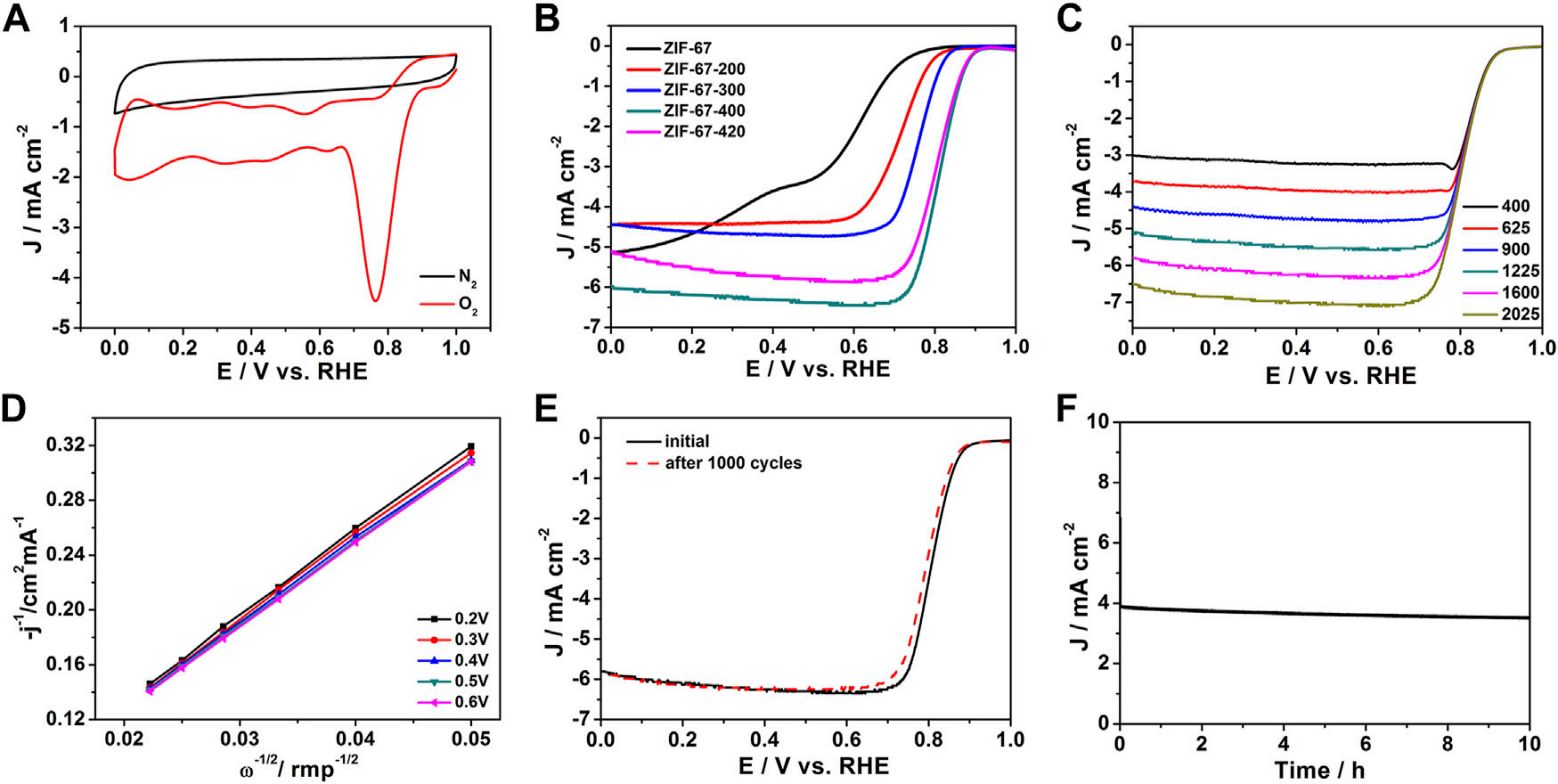
**(A)** Cyclic voltammograms (CV) of the ZIF-67-400 catalyst, **(B)** Linear sweep (LSV) diagrams of catalysts at different heating temperatures, **(C)** LSV curves of the ZIF-67-400 catalyst at different rotating speeds, **(D)** K-L curves of the ZIF-67-400 catalyst, **(E,F)** Cyclic stability diagrams and i-t diagrams of the ZIF-67-400 catalyst.

Figure 4A shows the cyclic voltammetry (CV) curve of ZIF-67-400 in the 0.1 M KOH electrolyte saturated with nitrogen and oxygen. In nitrogen, no redox peak was observed in the CV Curve, but a clear redox peak was observed in the O_2_ saturated solution. This result shows that the ZIF-67-400 catalyst has excellent catalytic performance for oxygen reduction in alkaline solutions. The LSV polarization curve at 1,600 rpm shown in Figure 4B further confirmed the excellent ORR activity of ZIF-67-400. In 0.1 M KOH solution, the ZIF-67-400 catalyst exhibits higher initial potential and half wave potential (E_onset_ = 0.91 V, E_1/2_ = 0.82 V) than ZIF-67 (E_onset_ = 0.78 V, E_1/2_ = 0.58 V), ZIF-67-200 (E_onset_ = 0.84 V, E_1/2_ = 0.71 V), ZIF-67-300 (E_onset_ = 0.89 V, E_1/2_ = 0.76 V), ZIF-67-380 (E_onset_ = 0.89 V, E_1/2_ = 0.80 V), and ZIF-67-420 (Eonset = 0.9 V, E_1/2_ = 0.80 V), so the order of ORR activities was ZIF-67 < ZIF-67-200 < ZIF-67-300 < ZIF-67-380 < ZIF-67-420 < ZIF-67-400, and the performance of the ZIF-67-400 catalyst is comparable to that of commercial Pt/C (E_onset_ = 0.96 V, E_1/2_ = 0.83 V). In addition, our ZIF-67-400 catalyst is superior to other ZIF-67 catalysts reported in literature, such as Zn-Co-ZIF/GO-920 (E_onset_ = 0.91 V, E_1/2_ = 0.80 V) (Zhu et al., 2019) and ZIF-67/PAN-800 (E_onset_ = 0.90 V, E_1/2_ = 0.81 V) (Guo et al., 2019b) and is comparable to some high-temperature pyrolysis materials (Table 1). It can be seen that ZIF-67 has the best catalytic performance at 400°C, which may be attributed to the change of surface groups, the decrease in size, and the gradual increase of local carbonization degree and defects.

**TABLE 1:** Recent reports on the ORR performance of MOF-based electrocatalysts in 0.1 M KOH.

MOFs/Precursors	ORR electrocatalyst	Half-wave potential (V)	Refs
Mn^2+^/ZIF-8	Mn_x_O_y_/N-C-3	0.871	Shah et al. (2018b)
Zn/Co-MOFs	^22.5^Co-N-C	0.91	Shah et al. (2017)
ZIF-67/CoAl-LDH	Co-CNTs/NS-CoO_x_AlO_y_	0.835	Najam et al. (2019)
ZnCo-MOF@PDAP	ZnCo-PNC	0.91	Najam et al. (2022)
ZIF-67	P-Co-CNTs	0.887	Shah et al. (2021b)
ZIF-67	ZIF-67-400	0.82	This work
Zn-Co-ZIF/GO	Zn-Co-ZIF/GO-920	0.807	Zhu et al. (2019)
ZIF-67/PAN	ZIF-67/PAN-800	0.81	Guo et al. (2019b)

In addition, the LSV polarization curves of ZIF-67-400 at different rotational speeds (400–2025 rpm) were tested, and the ORR catalytic mechanism was discussed. It can be seen from Figure 4C that with the increase in rotating speed, the limiting current density gradually increases. This is because the increase in rotating speed accelerates the diffusion speed and diffusion range of oxygen so that the catalyst surface can be more fully contacted with oxygen, thus speeding up the reaction rate. The electron transfer number of the catalyst was calculated according to the LSV polarization curve at different rotational speeds. As shown in Figure 4D, the electron transfer number at 0.2–0.6 V was calculated to be 4.1, which indicates the ORR reaction is a four-electron transfer reaction. The RRDE result in Supplementary Figure S2 showed that the average electron transfer number of ZIF-67-400 is 3.9, and the H_2_O_2_ yield is below 10%, which is consistent with the electron transfer number from K–L plots.

As shown in Figure 4E, the ORR stability test of ZIF-67-400 exhibited that after 1,000 cycles in 0.1 M KOH, the initial potential and half-wave potential have only slight attenuation. Under the condition of a fixed voltage of 0.7 V, the long-term stability of the catalyst by chronoamperometry further proves that ZIF-67-400 can maintain 96% activity after 10 h of long-term operation (Figure 4F). These results show that ZIF-67–400 has excellent durability.

## 4 Conclusion

In conclusion, a facile low-temperature calcination route to modify the ORR activity of ZIF-67 is proposed. The controlled heating temperature selectively produces local defects and at the same time retains the overall skeleton structure. The intrinsic Co-N_4_ metal sites are activated by the forming of the defects, and the ORR electrocatalytic performance is improved with the half-wave potential of 0.82 V and 1,000 cycle stability for ZIF-67-400. The local defects are caused by partial deligandation of the skeleton and partial destruction of the coordination bond of Co-N_4_. It may offer a new direction for the modification of the MOF structure and catalytic activity for energy conversion and storage.

## Data Availability

The original contributions presented in the study are included in the article/Supplementary Material; further inquiries can be directed to the corresponding author.
