# Supplementary material for: Partial deligandation activated ZIF-67 for efficient electrocatalytic oxygen reduction reaction
Source: Front Chem. 2022 Oct 6;10:983549. doi: 10.3389/fchem.2022.983549 (PMC9583129; doi:10.3389/fchem.2022.983549)
Supplement: Supplementary file 1 [file DataSheet1.doc]

Supporting Information


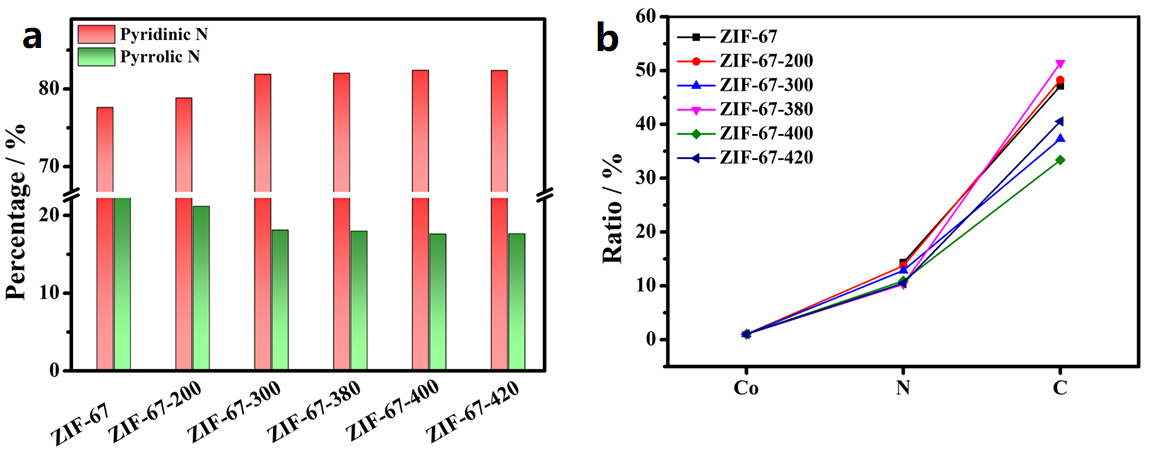


Fig. S1 The percentage composition of different N species (a) and the Co, N, C ratio (b) in ZIF-67 at different temperature.


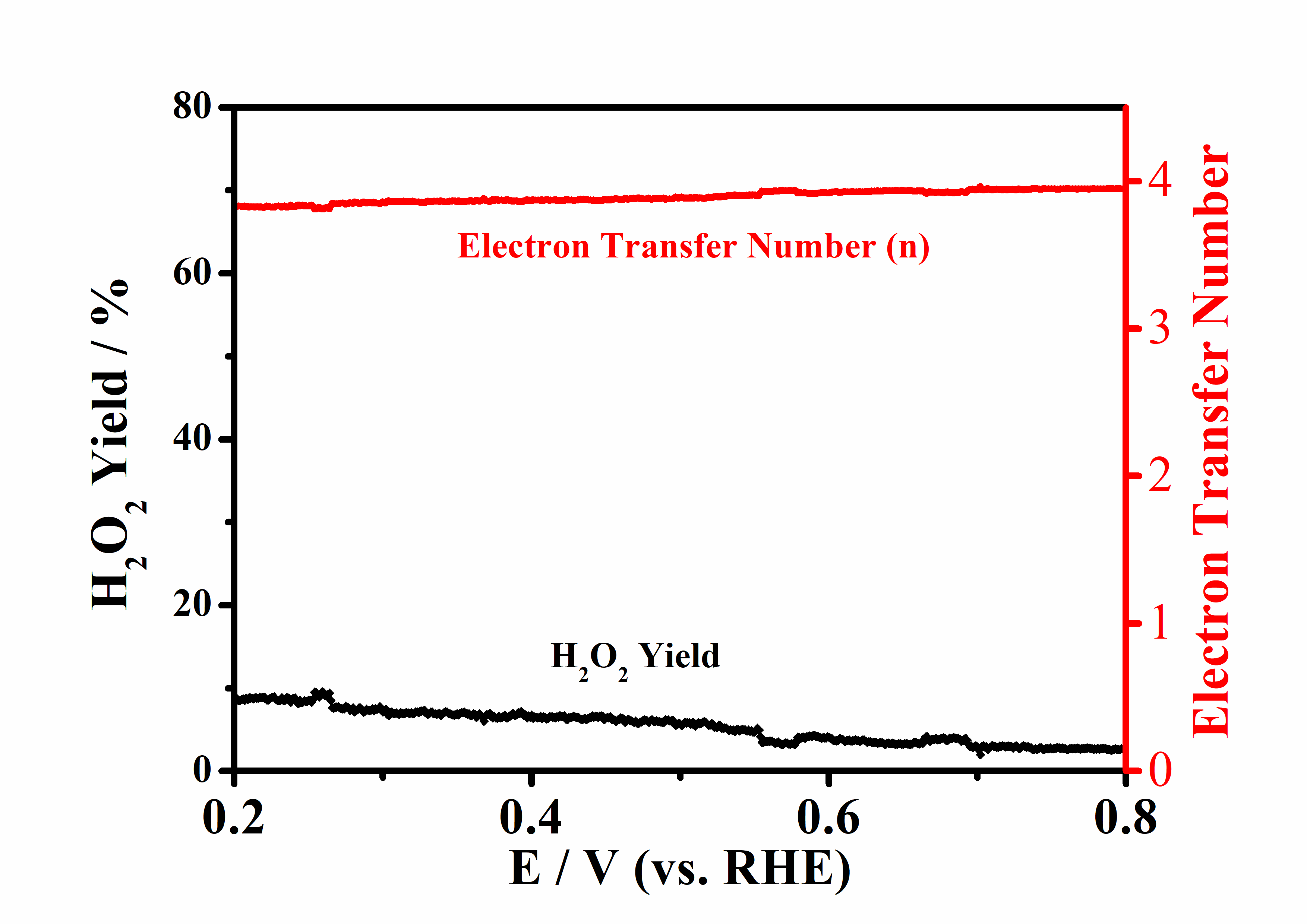


Fig. S2 Electron transfer number (n) and H_2_O_2_ yield of ZIF-67-400.
